# Gasdermin D-dependent neutrophil extracellular traps exacerbate cytokine storm contributing to pyoderma gangrenosum pathogenesis

**DOI:** 10.1016/j.isci.2025.111925

**Published:** 2025-01-30

**Authors:** Sheng Li, Shuni Ying, Hong Fang, Jianjun Qiao

**Affiliations:** 1 Department of Dermatology, The First Affiliated Hospital, Zhejiang University School of Medicine, Hangzhou, Zhejiang, China

**Keywords:** molecular biology, immunology

## Abstract

Pyoderma gangrenosum (PG) is characterized by the agonizing necrotizing ulcers with non-infectious neutrophil infiltration. Neutrophil extracellular traps (NETs) represent one of the mechanisms of neutrophils activation, and gasdermin D (GSDMD) plays a regulatory role in NETs. In this study, we discovered that the serum levels of NETs were elevated in PG patients compared to healthy controls. Injection of serum from PG patients into the dorsal skin of wild-type mice led to the formation of localized cutaneous ulcers. Furthermore, subsequent modeling demonstrated a significant increase of NETs and GSDMD in skin lesions and peripheral blood serum of wild-type mice. In *GSDMD*^*−/−*^ mice, the severity of skin ulcers after modeling was significantly diminished. Overall, our findings shed light on the role of GSDMD in regulating the production of NETs by neutrophils and the release of inflammatory factors in the pathogenesis of PG and establish an animal model for studying PG.

## Highlights

• NETs formation involved in the pathogenesis of PG
• The serum of patients with PG can induce PG-like ulcers on the backs of mice
• GSDMD contributes to the pathogenesis of PG by regulating NETs production

## Graphical abstract

**Figure:**
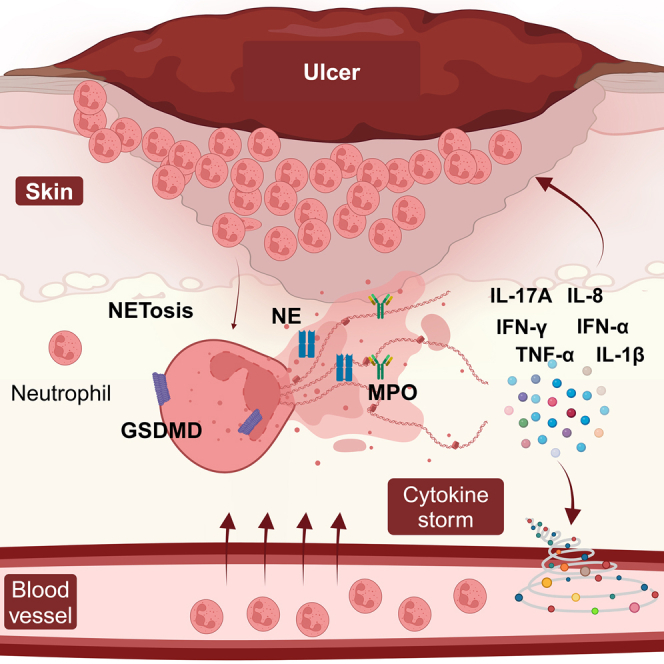

## Introduction

Pyoderma gangrenosum (PG) is a rare inflammatory skin disease with typical skin lesions characterized as rapid progressive relapsing necrotic ulcers accompanied by severe pain.^1^ The first case of PG was reported by Brocq in 1908, and, since then, clinical physicians from various countries have reported relevant cases. In 1930, Brunsting et al. summarized PG more completely for the first time,^2^ proposing that the characteristic manifestations of PG were papules, vesicles, pustules, or nodules that subsequently formed creeping, painful ulcers and necrosis with purple-red raised margins, progressing rapidly. Skin lesions occur mainly on the lower limbs and buttocks and can recur. Patients often develop the disease after trauma or iatrogenic injury, known as pathergic reaction.^3^ PG patients often have systemic diseases such as inflammatory bowel disease, hematological system diseases, and arthritis. Additional monogenic autoinflammatory syndromes, such as pyogenic arthritis, pyoderma gangrenosum and acne (PAPA) syndrome, pyoderma gangrenosum, acne and hidradenitis suppurativa (PASH) syndrome, and pyogenic arthritis, pyoderma gangrenosum, acne and hidradenitis suppurativa (PAPASH) syndrome, can also be classified as types of syndromic PG. This is because they exhibit lesions that closely mimic those seen in classic ulcerative PG. Initially, PG was thought to be an infectious disease and was later corrected to be an inflammatory skin disease. Moreover, PG can manifest as a paradoxical reaction; the debate remains open regarding whether PG is a reaction to medication or a dermatological symptom of an underlying condition.^4^

This disease has genetic susceptibility, and genes related to the onset of PG reported in the literature include *PTPN6*, *PSTPIP1*, *MEFV*, *NLRP3*, *NLRP12*, *LPIN2*, and *NOD2*, among others.^5^^,^^6^^,^^7^^,^^8^^,^^9^^,^^10^ There are also reports of familial cases.^11^^,^^12^^,^^13^^,^^14^^,^^15^ In addition to genetic factors, trauma is the most common cause of PG. Henry et al. found that tissue damage could induce interleukin (IL)-36-mediated inflammatory responses by releasing corneal keratinocyte after tissue damage,^16^ and IL-36 is one of the important inflammatory cytokines in the pathogenesis of PG. Moreover, trauma is also involved in the onset of PG by releasing IL-8 and autoantigens.^17^^,^^18^ Inflammatory factors such as IL-36 released by trauma further recruit and activate neutrophils, promoting the release of more inflammatory factors by neutrophils, corneal keratinocytes, and other cells, thus activating and exacerbating inflammatory responses.^19^^,^^20^^,^^21^ At the same time, genes such as *PSTPIP1*, *MEFV*, *NLRP3*, and *NLRP12*, which are abnormally expressed, lead to an increase in the release of IL-1β, promoting the further release of inflammation-related cytokines associated with neutrophils, thereby continuing to recruit and activate neutrophils.^22^^,^^23^^,^^24^ Moreover, the pathways involved in the recruitment and activation of neutrophils, leading to the inflammatory response, include the release of IL-17E by keratinocytes, which promotes macrophages to secrete CXCL-1 and CXCL-10, as well as CCL-20.^25^^,^^26^ Th1 and Th17 cells promote the secretion of CCL-3, CCL-5, IL-17A, and interferon (IFN)-γ.^27^ IL-17A works in concert with inflammatory factors such as tumor necrosis factor alpha (TNF-α) and IL-1β, further facilitating the accumulation of monocytes and neutrophils, thereby intensifying the inflammatory response.^28^ These findings suggest that various factors recruit and activate neutrophils, thus promoting the secretion of inflammatory cytokines and activating inflammatory responses, which are key mechanisms in PG pathogenesis. However, it is still unclear how the massive accumulation of activated neutrophils occurs, its regulatory mechanism, and how it exerts its pathogenic role, which is also the critical scientific issue addressed in this study.

Neutrophils are one of the effectors of innate immunity in the body’s defense system during physiological conditions. They aggregate and activate when the body is infected or has aseptic inflammation, playing a protective role.^29^^,^^30^ However, abnormally aggregated and activated neutrophils can exert their pathogenic effects through phagocytosis, degranulation, and the generation of neutrophil extracellular traps (NETs), especially in the occurrence of various inflammatory skin diseases.^31^^,^^32^^,^^33^^,^^34^ The core feature of NETs is a net-like structure composed of deoxyribonucleic acid (DNA) escaping from neutrophils, attached to various proteins, such as histones, granulins, and cytoplasmic proteins. This structure can effectively capture invading microorganisms, and the process of neutrophils producing NETs is called NETosis.^35^^,^^36^ Gasdermin D (GSDMD) is a member of the gasdermin protein family, which plays a pivotal role in pyroptosis, an inflammatory form of cell death intricately linked to immune responses. GSDMD is present in cells as an inactive precursor. When cells are stimulated by pathogens or danger signals, specific inflammatory caspases, such as caspase-1 or caspase-11, are activated and cleave GSDMD, producing an active N-terminal fragment (GSDMD-N). Further research has revealed that GSDMD also plays a role in the generation of NETs. The pores formed by GSDMD facilitate the release of neutrophil elastase (NE) and myeloperoxidase (MPO), which are involved in the early stages of NETosis.^37^^,^^38^^,^^39^ NE further activates GSDMD, contributing to the formation of pores in the neutrophil membrane. This process leads to the release of decondensed chromatin and granule-bound proteins from the cell, thereby promoting NET formation.

Therefore, this study aimed to detect the expression levels of inflammatory factors in the skin lesions and serum of PG patients and used GSDMD^−/−^ mice and wild-type (WT) mice to establish a mouse model of PG, measuring the expression levels of inflammatory factors in the skin lesions, peripheral blood, and serum, exploring the mechanism of the role of GSDMD-mediated NETs releasing inflammatory factors in ulcer formation and the pathogenesis of PG.

## Results

### NETs are enriched in circulating neutrophils and lesions from PG patients

**Figure 1:**
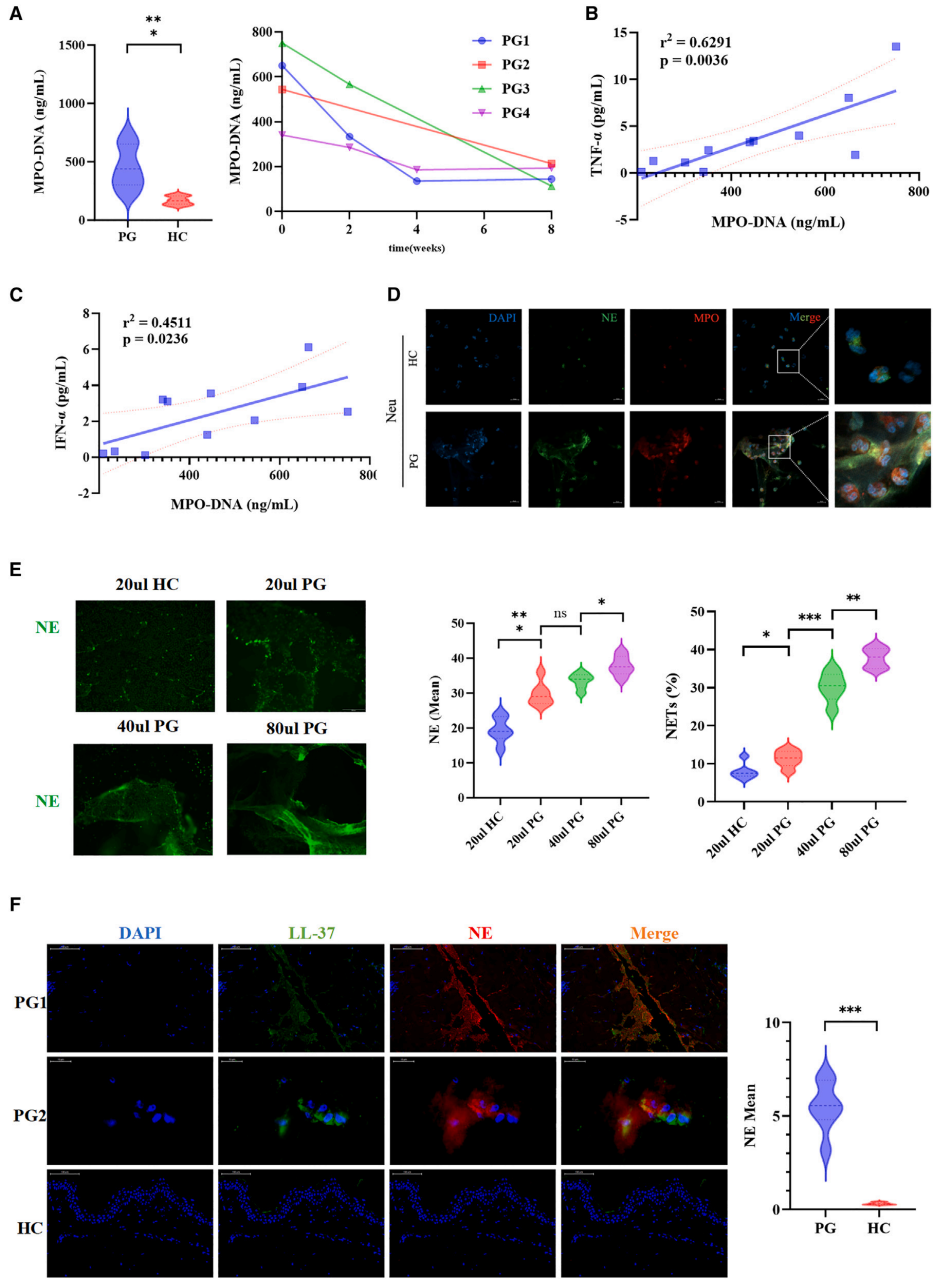
NET formation is increased in the circulating neutrophils and lesions from PG patients (A) Serum levels of MPO-DNA complexes in PG patients compared to those in HC and longitudinal changes prior to and after treatment in four representative patients with PG. (B and C) The serum levels of MPO-DNA complexes positively correlated with TNF-α and IFN-α in patients with PG. All patients with PG were included in the calculation of Spearman’s rank correlation coefficient. (D) Representative immunofluorescence staining of the serum of PG-induced NETs formation (DNA: blue; NE: green; MPO: red) in the neutrophils from HC. (E) Representative immunofluorescence staining and proportion of NETs stimulated by the serum of healthy control and PG (20, 40, and 80 μL) in the neutrophils from healthy donors. (F) Immunofluorescent microscopy images detected the NETosis in the skin lesions of PG patients and HC. Median and error bars representing interquartile range are displayed in (A), (E), and (F). PG, pyoderma gangrenosum; NETs, neutrophil extracellular traps; HC, healthy control; MPO, myeloperoxidase; NE, neutrophil elastase. ∗*p* < 0.05, ∗∗*p* < 0.01, and ∗∗∗*p* < 0.001.

In order to clarify the correlation between the NETs and the onset of PG, this study detected the NETs and inflammatory factors in the serum of PG patients and analyzed their correlation. The ability of neutrophils from peripheral blood of PG patients to spontaneously produce NETs was also evaluated. Furthermore, the ability of PG patient serum to induce NETs production in neutrophils from heathy control (HC) was examined. ELISA was used to measure the expression levels of MPO-DNA complexes in the serum of PG patients. It was found that the expression levels of MPO-DNA complexes in the serum of PG patients were significantly higher than those in HC (*p* < 0.001). The expression levels of MPO-DNA complexes in the serum of PG patients were significantly decreased after effective treatment, as observed by ELISA (Figure 1A). Effective treatment means that, after the patient receives systemic corticosteroid therapy, their condition (including the size of ulcers, the degree of pain, and abnormal laboratory test results) shows significant improvement, achieving the therapeutic effect. It was found that the expression levels of IL-1β (*p* < 0.001), IL-8 (*p* < 0.001), TNF-α (*p* < 0.01), IL-17A (*p* < 0.05), IFN-γ (*p* < 0.01), and IFN-α (*p* < 0.01) in the serum of PG patients were significantly higher than those in HC (Figure S1A). Correlation analysis was performed to examine the relationship between the expression levels of MPO-DNA complexes in the serum of PG patients and the expression levels of IL-1β, IL-8, IL-17A, IL-6, IL-10, TNF-α, IFN-γ, and IFN-α in the serum. It was observed that the expression levels of MPO-DNA complexes in the serum of PG patients were positively correlated with the expression levels of TNF-α (r2 = 0.6291, *p* = 0.0036) and IFN-α (r2 = 0.4511, *p* = 0.0236) in the serum (Figures 1B and 1C).

To evaluate the capacity of serum from PG patients to induce NETosis in neutrophils, immunofluorescence analysis was conducted to measure the NETs in neutrophils from HC after stimulation with serum from PG patients. The results showed that the NETs in neutrophils stimulated with PG patient serum were higher compared to those stimulated with HC serum (Figure 1D). Additionally, the NETs increased in tandem with the concentration of PG patient serum (Figure 1E). In order to ascertain whether NETosis occurs in neutrophils from the peripheral blood of PG patients, immunofluorescence staining was employed to evaluate the NETs in neutrophils extracted from the peripheral blood of both PG patients and HC. It was observed that the NETs in neutrophils extracted from the peripheral blood of PG patients were significantly higher (*p* < 0.001) compared to HC (Figure S1B).

Immunofluorescence analysis was employed to assess the NETs in skin lesions of PG patients. It was observed that the proportion of neutrophils undergoing NETosis in the skin lesions of PG patients was significantly higher than that of HC (Figure 1F).

### Serum from patients with PG can induce PG-like manifestations in mice

**Figure 2:**
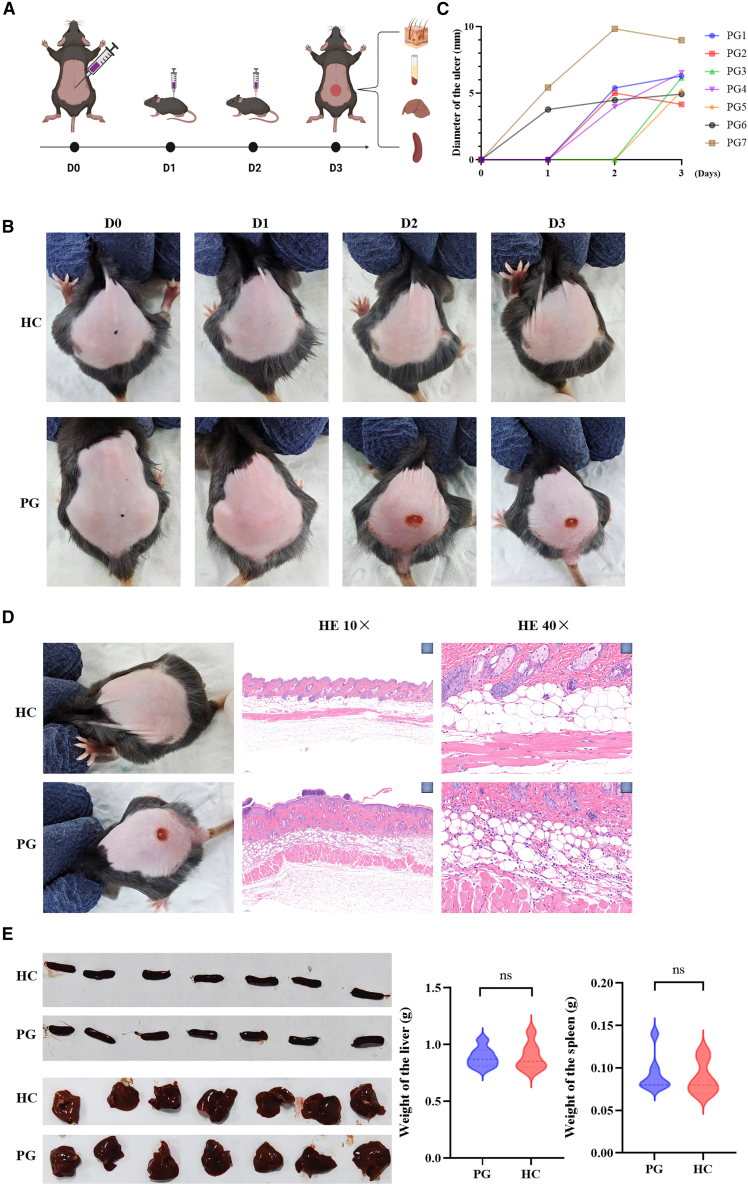
Serum from patients with PG can induce PG-like manifestations in mice (A) Subcutaneously injecting the serum of PG patients into the dorsal area of WT mice to induce ulcer. (B and C) The ulcers induced by injecting the serum of PG patients. (D) The histopathological examination of the skin lesions in the PG group compared to the HC group. (E) The differences between the PG group and the HC group in terms of liver size, spleen size, liver weight, and spleen weight in mice. PG, pyoderma gangrenosum; WT, wild-type; HC, healthy control.

By subcutaneously injecting the serum of PG patients into the dorsal area of WT mice, it was observed that the mice in the PG group developed purple-red ulcers on their backs with clear borders (Figure 2A). The surrounding skin exhibited edema, and some ulcer centers showed necrosis. The ulcers were located at the injection site where a raised skin mound formed. As the modeling time increased, the color of the ulcers deepened, and the skin around some ulcers slightly raised, with a depressed center and wrinkling of the surrounding normal skin. In contrast, the mice in the HC group had normal-looking skin at the injection site and did not develop any ulcers (Figures 2B and 2C). The skin lesions of mice were subjected to H&E staining. The histopathological examination of the skin lesions in the PG group revealed the presence of microabscesses formed by the aggregation of neutrophils in the epidermis. Neutrophil infiltration was observed around blood vessels in the dermal layer, accompanied by vascular dilation and extravasation of red blood cells. The adipose layer showed significant infiltration of neutrophils, displaying changes resembling PG (Figure 2D). No significant differences were observed between the PG group and the HC group in terms of liver size, spleen size, liver weight, and spleen weight in mice (Figure 2E).

### Elevation of NETs and related inflammatory factor expression levels in a mouse model of PG

**Figure 3:**
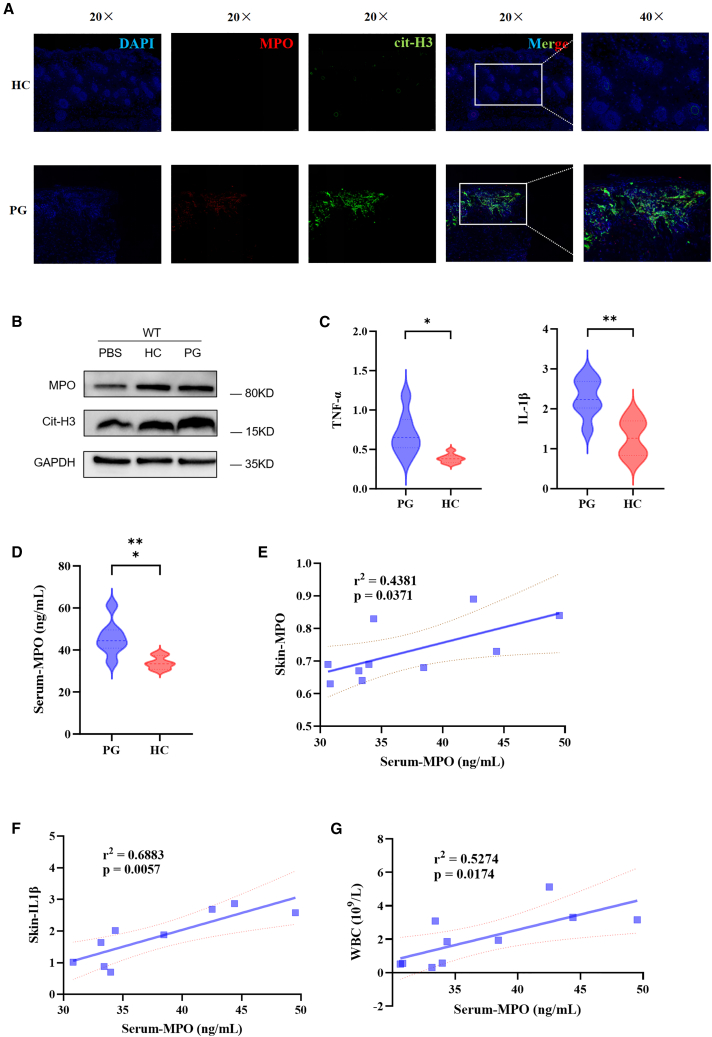
Elevation of NETs and related inflammatory factor expression levels in a mouse model of PG (A) Representative immunofluorescence staining of the expression level of MPO and cit-H3 in skin lesions of PG group mice compared to HC group mice (DNA: blue; cit-H3: green; MPO: red). (B) The levels of MPO and cit-H3 expression in the skin lesions of PG group mice compared to HC group mice and PBS group mice with WB methods. (C) The expression levels of IL-1β and TNF-α in the skin lesions of PG group mice compared to HC group mice. (D) The expression levels of MPO in the serum of PG group mice compared to HC group mice. (E–G) The correlation between the MPO levels in the serum of the PG group and HC group mice and laboratory indicators and serum inflammatory factors. PG, pyoderma gangrenosum; HC, healthy control; NETs, neutrophil extracellular traps; MPO, myeloperoxidase; cit-H3, citrullinated histone; IL, interleukin; TNF, tumor necrosis factor. ∗*p* < 0.05, ∗∗*p* < 0.01, and ∗∗∗*p* < 0.001.

The expression levels of MPO in skin lesions of PG group mice and skin tissues of HC group mice were detected using immunofluorescence. It was found that the NETs in skin lesions of PG group mice were significantly higher than those of HC group (Figure 3A). The levels of MPO and citrullinated histone (cit-H3) expression were detected in the skin lesions of PG group mice compared to HC group mice and PBS group mice (Figure 3B). Using quantitative reverse-transcription PCR (RT-qPCR), the expression levels of MPO, C-reactive protein, IL-1β, IL-6, IL-8, IFN-γ, and TNF-α were detected in the skin lesions of the PG group mice and the skin tissues of the HC group mice. It was found that the expression levels of IL-1β (*p* < 0.01) and TNF-α (*p* < 0.05) in the skin lesions of the PG group mice were significantly higher than those in the skin tissues of the HC group mice (Figure 3C). The expression levels of MPO in the serum of the PG group and HC group mice were detected using ELISA. It was found that the MPO expression level in the serum of the PG group mice (*p* = 0.022) was significantly higher than that in the HC group mice (Figure 3D). Correlation analysis was performed between the MPO levels in the serum of the PG group and HC group mice and laboratory indicators and serum inflammatory factors. It was found that the expression level of MPO in the serum of mice was positively correlated with the expression level of MPO in the skin lesions (r2 = 0.4381, *p* = 0.0371) (Figure 3E). The expression level of MPO in the serum was positively correlated with the expression level of IL-1β in the skin lesions (r2 = 0.6883, *p* = 0.0057) (Figure 3F), and the expression level of MPO in the serum was positively correlated with the peripheral blood leukocyte count (r2 = 0.5274, *p* = 0.0174) (Figure 3G).

### The expression of GSDMD is upregulated in the serum and skin lesions of PG patients and PG mouse model

**Figure 4:**
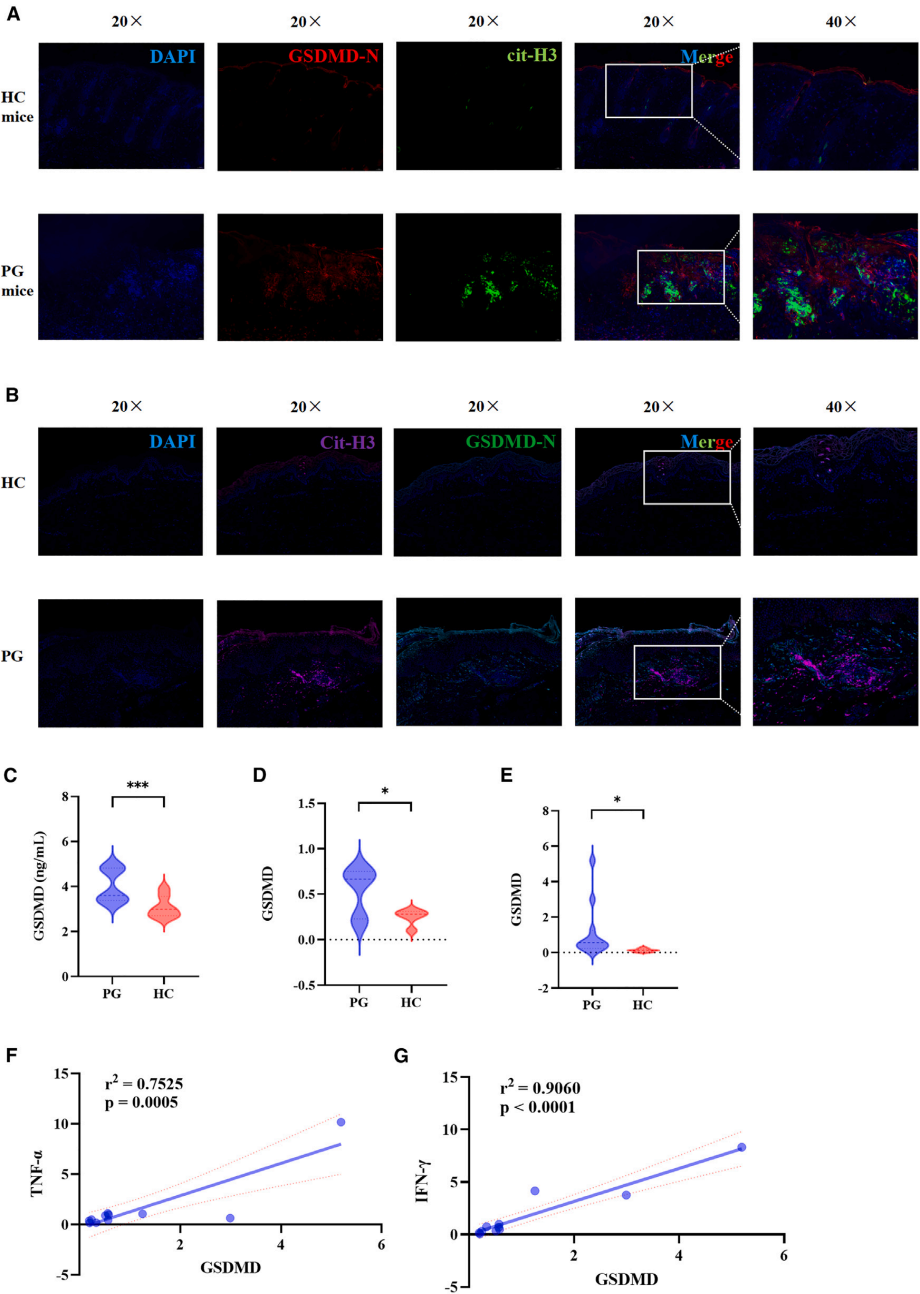
The expression of GSDMD in the serum and skin lesions of PG patients and PG mouse model (A) Representative immunofluorescence staining of the expression level of GSDMD-N and cit-H3 in skin lesions of PG group mice compared to HC group mice (DNA: blue; cit-H3: green; GSDMD-N: red). (B) Representative immunofluorescence staining of the expression level of GSDMD-N and cit-H3 in skin lesions of PG group mice compared to HC group mice (DNA: blue; GSDMD -N: green; GSDMD-N: purple). (C) The expression level of GSDMD in the serum of PG mice and HC. (D) The expression level of GSDMD in the skin lesions of PG mice and HC. (E) The expression level of GSDMD in the serum of PG patients compared to HC. (F and G) The correlation of the expression level of GSDMD in the serum of PG patients and the expression level of TNF-α and IFN-γ. PG, pyoderma gangrenosum; HC, healthy control; GSDMD-N, gasdermin D N-terminal domain; TNF, tumor necrosis factor; IFN, interferon; cit-H3, citrullinated histone. ∗*p* < 0.05, ∗∗*p* < 0.01, and ∗∗∗*p* < 0.001.

The expression levels of GSDMD-N and cit-H3 in skin lesions of PG group mice and skin tissues of HC group mice were detected using immunofluorescence. It was found that the expression levels of GSDMD-N and cit-H3 in skin lesions of PG group mice were significantly higher than those of the HC group (Figure 4A). Also, the expression levels of GSDMD-N and cit-H3 in skin lesions of PG patients were significantly higher than those of HC (Figure 4B). ELISA was used to measure the expression level of GSDMD in the serum of PG mice. It was found that the expression level of GSDMD in the serum of PG mice (*p* = 0.022) was significantly higher than that in the HC (Figure 4C). RT-qPCR was used to measure the expression level of GSDMD in the skin lesions of PG mice and the skin tissues of HC mice. It was found that the expression level of GSDMD in the skin lesions of PG mice (*p* = 0.022) was significantly higher than that in the skin tissues of HC mice (Figure 4D). The expression levels of GSDMD, IFN-γ, TNF-α, and IL-17A in the serum of PG patients were detected using RT-qPCR, and their correlations were analyzed. It was found that the expression level of GSDMD in the serum of PG patients was significantly higher than that in HC (Figure 4E). The expression level of GSDMD in the serum of PG patients was positively correlated with the expression level of TNF-α in the serum (r2 = 0.7525, *p* = 0.0005) (Figure 4F). The expression level of GSDMD in the serum of PG patients was also positively correlated with the expression level of IFN-γ in the serum (r2 = 0.9060, *p* < 0.0001) (Figure 4G). Moreover, the expression level of GSDMD in the serum of PG patients showed a positive correlation with the expression level of IL-17A in the serum (r2 = 0.6914, *p* = 0.0015) (Figure S2A).

### GSDMD can alleviate systemic inflammation and cutaneous lesions in PG-like mice

**Figure 5:**
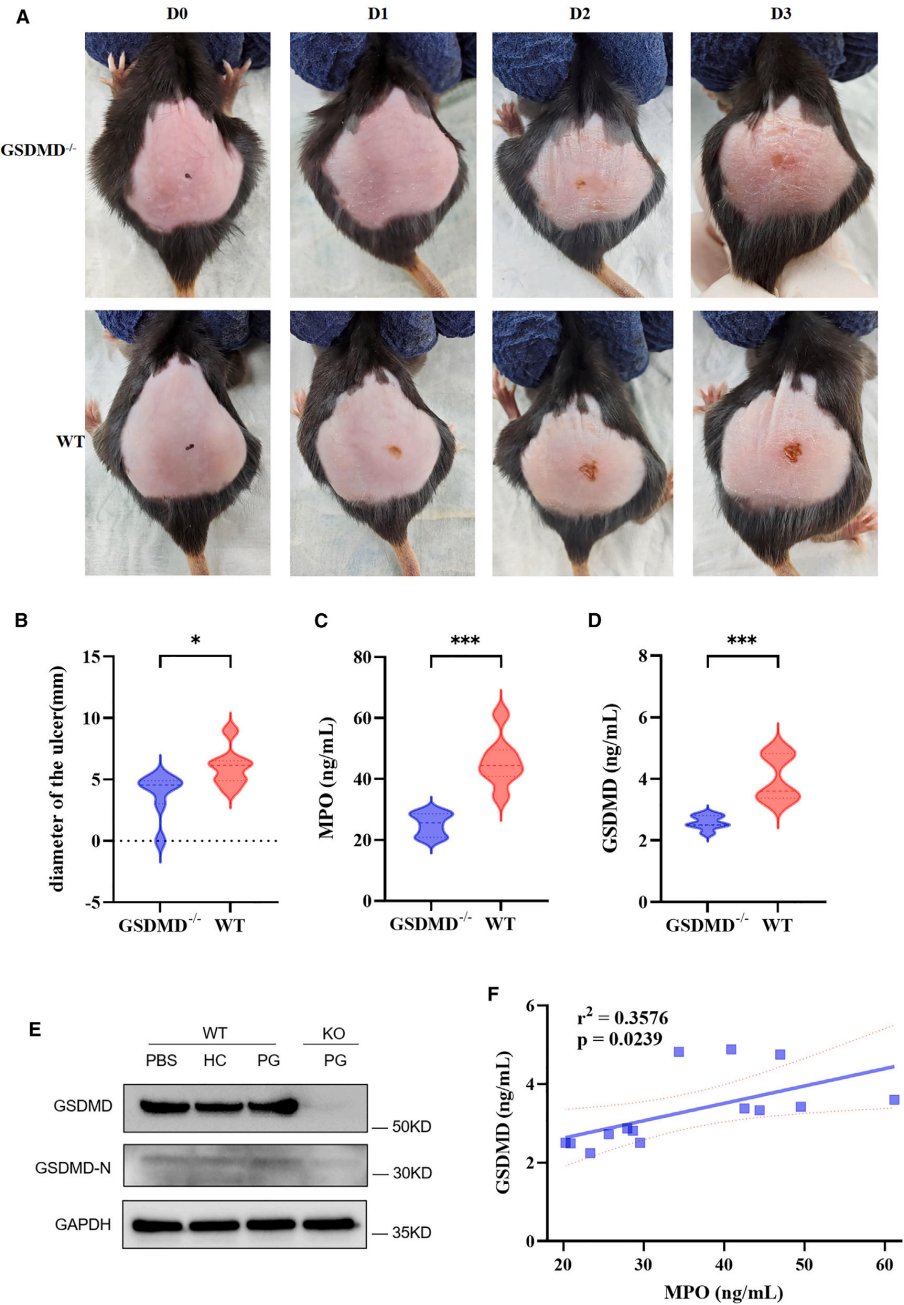
GSDMD can alleviate systemic inflammation and cutaneous lesions in PG-like mice (A) The difference of PG-like ulcers between *GSDMD*^*−/−*^ mice and WT mice in the modeled groups. (B) The diameter of the skin lesions in the *GSDMD*^*−/−*^ group mice compared to WT mice group. (C–E) The levels of GSDMD and GSDMD-N expression in the serum of PG group and HC group with *GSDMD*^*−/−*^ and WT mice. (F) The correlation between the expression levels of MPO and GSDMD in mouse serum. PG, pyoderma gangrenosum; WT, wild-type; GSDMD, gasdermin D; MPO, myeloperoxidase. ∗*p* < 0.05, ∗∗*p* < 0.01, and ∗∗∗*p* < 0.001.

A PG mouse model was established by subcutaneous injection of diluted serum from PG patients into *GSDMD*^*−/−*^ mice and WT mice on the back. It was found that both *GSDMD*^*−/−*^ mice and WT mice in the modeled groups developed PG-like ulcers on their backs. However, compared with the WT group, the *GSDMD*^*−/−*^ group exhibited reduced ulcer area, lighter color, unclear boundaries, and no necrosis in the center (Figure 5A). The diameter of the skin lesions in the *GSDMD*^*−/−*^ group mice was significantly smaller than that in the WT group (Figure 5B). Skin lesions in the *GSDMD*^*−/−*^ group mice appeared within the first 1–3 days after the first injection of PG patient serum. The lesions reached their maximum size on the second and third days and then remained stable or slightly decreased in size. The back skin lesions of GSDMD^−/−^ and WT mice were subjected to H&E staining. It was observed that the histopathological features of the skin lesions in *GSDMD*^*−/−*^ mice showed relatively normal epidermis, with a small amount of neutrophil infiltration in the dermis and adipose layer (Figures S3A and S3B). Moreover, the degree of neutrophil infiltration was significantly milder compared to the WT group (Figure 5C). The levels of GSDMD-N and GSDMD expression were detected in the serum of *GSDMD*^*−/−*^ and WT mice groups using ELISA and Western Blot (WB) methods. The analysis of their correlation revealed that the expression levels of GSDMD-N and GSDMD in the serum of *GSDMD*^*−/−*^ group were significantly lower compared to the WT group (Figures 5D and 5E). Furthermore, a positive correlation was observed between the expression levels of MPO and GSDMD in mouse serum (r2 = 0.3576, *p* = 0.0239) (Figure 5F).

## Discussion

PG is one of the most representative diseases in neutrophilic dermatoses, characterized by non-infectious neutrophil infiltration. However, the activated form of neutrophil aggregation and its role in the pathogenesis of PG remain unclear, and there is a lack of animal models for studying PG. In this study, we found that the expression levels of inflammatory factors in the serum of PG patients were significantly increased. The spontaneous production of NETs by peripheral blood neutrophils in PG patients was significantly enhanced. Furthermore, the ability of neutrophils to produce NETs in response to serum stimulation from PG patients was also significantly increased. Additionally, the NETs in PG skin lesions were significantly elevated. Moreover, this study successfully established a PG mouse model and found that GSDMD is involved in regulating NET generation and affects disease pathogenesis.

Abnormal expression of NETs is associated with the pathogenesis of various skin diseases. The former studies of NETs in PG have some limitations including a lack of quantitative analysis of NETs in PG patient serum and correlation analysis with inflammatory factors, a lack of investigation into whether PG patient serum can induce NETs production in HC neutrophils, and a lack of correlation analysis between NETs expression levels and disease severity.^40^^,^^41^^,^^42^ Our study found elevated levels of NETs expression in PG serum, which positively correlated with levels of TNF-α and IFN-α in PG patient serum. This suggests that NETs can serve as indicators of changes in PG condition and facilitate treatment management for PG patients. Furthermore, PG patient serum was able to induce NETs production in peripheral blood neutrophils from HC, and the occurrence of NETs increased with higher concentrations of PG patient serum. This indicates the presence of crucial pathogenic factors in PG patient serum that can stimulate NETs production in peripheral blood neutrophils, leading to disease onset. The mechanism behind this phenomenon needs further exploration in the next section. Additionally, the proportion of activated cells among neutrophils aggregated in PG skin lesions was higher than that in HC, and the proportion and expression levels of neutrophil-produced NETs were also higher in PG. This suggests that the neutrophils accumulating in PG skin lesions likely participate in lesion formation through NETs production, thus contributing to the pathogenesis of PG.

Research has found that the construction of *PTPN6*^*spin*^ mice leads to spontaneous inflammatory changes in the foot pads of mice.^43^^,^^44^^,^^45^ However, *PTPN6*^*spin*^ mice also have many limitations, such as the inability to simulate the clinical manifestations of PG patients well, especially the typical skin ulcers. In addition, IL-1α pathway expression is abnormal in *PTPN6*^*spin*^ mice, but it does not involve inflammatory factors such as IL-36, IL-8, IL-1β, IL-17A, and TNF-α, which are associated with the onset of PG. Therefore, it cannot simulate the inflammatory response in PG patients well. In order to construct an animal model similar to PG, this study induced PG-like ulcers on the back of mice by subcutaneous injection of PG patient serum. After injection of PG patient serum into the back of WT mice, skin ulcers appeared and were characterized by clearly defined purple-red ulcers, edema around the edges of the skin, partial necrosis in the center of some ulcers. The pathological features of the skin lesions included focal serous exudation in the epidermal layer, localized neutrophil infiltration, neutrophil infiltration around blood vessels in the dermal layer, vasodilation, extravasation of red blood cells, and significant infiltration of neutrophils in the adipose layer. These pathological features are very similar to those of PG. In addition, the expression levels of IL-1β and TNF-α in the skin lesions of PG mice were found to be significantly elevated. Peripheral blood leukocyte count and neutrophil count also increased significantly, which are similar to the abnormal laboratory indicators of PG patients. In conclusion, the PG mouse model constructed in this study can better simulate the clinical characteristics and pathological changes of PG patients, providing assistance for further research on the pathogenesis of PG.

The findings of this study indicate an abnormal elevation in the expression levels of NETs in the skin lesions and serum of both PG patients and PG mice. However, the specific regulatory and pathogenic mechanisms of NETs in PG remain unclear. The regulatory mechanisms underlying NETs formation are exceedingly complex. Following neutrophil aggregation, various endogenous and exogenous stimuli such as microorganisms, immune complexes, cytokines, and inflammatory corpuscles collectively induce neutrophils to release NETs through multiple pathways involving calcium channels, protein kinase C, Janus kinase2, and others.^46^ In recent years, it has been discovered that GSDMD also participates in the generation of NETs.^37^^,^^38^^,^^47^ In this study, elevated levels of GSDMD expression were observed in both the skin lesions and serum of PG mice. Additionally, a PG model was established in *GSDMD*^*−/−*^ mice, which exhibited reduced area, lighter color, unclear boundaries, and absence of central necrosis in the PG-like ulcerations. Histological analysis of the skin lesions showed a significant decrease in neutrophil infiltration in *GSDMD*^*−/−*^ mice. Furthermore, the NETs in the skin lesions and serum of PG models constructed in *GSDMD*^*−/−*^ mice were decreased compared to WT mice, with a positive correlation between GSDMD expression levels and NETs expression levels in serum. This suggests that GSDMD exerts its pathogenic effect by regulating the production of NETs by neutrophils. Lastly, a PG model constructed in *GSDMD*^*−/−*^ mice exhibited decreased expression levels of serum IFN-γ, IL-17A, and IL-1β compared to WT mice. Overall, knocking out GSDMD effectively alleviated the clinical symptoms, pathological changes, and response in mice with PG models, indicating that GSDMD may induce inflammatory reactions in peripheral blood, skin, and other tissues by regulating the production of NETs by neutrophils, leading to the manifestation of skin ulcers.

This study found elevated expression levels of GSDMD, IFN-γ, IL-17A, and TNF-α in the serum of PG patients and a positive correlation between GSDMD expression levels and the expression levels of IFN-γ, IL-17A, and TNF-α. GSDMD expression in PG skin lesions was significantly increased and partially overlapped with the expression of NETs. Moreover, cell experiments showed that the NETs released by *GSDMD*^*−/−*^ neutrophils were significantly lower compared to WT neutrophils when stimulated with PG patient serum.

In summary, this work uncovers that GSDMD mediates the production of NETs by neutrophils, leading to the release of inflammatory factors and participating in the pathogenesis of PG.

### Limitations of the study

The PG samples collected in this study originated from a single institution; thus, the sample size was limited. Future studies could conduct multicenter research to increase the sample size. The mechanism by which PG serum causes ulcers in mice has not been thoroughly investigated and warrants further in-depth research.

## Resource availability

### Lead contact

For further information and resource requests, please contact the lead contact, Jianjun Qiao (qiaojianjun@zju.edu.cn), who will facilitate these requests.

### Materials availability

All unique or stable reagents developed in this study are available from the lead contact upon completion of a material transfer agreement.

### Data and code availability

Data: The lead contact will provide all data reported in this article upon request. Additional information necessary to reanalyze the reported data can also be obtained from the lead contact.

Code: This article does not include original code.

## Acknowledgments

This work was supported by the 10.13039/501100001809National Natural Science Foundation of China (82273548 to H.F. and 82173400 to J.Q.).

## Author contributions

S.L.: methodology, formal analysis, and writing – original draft. S.Y.: formal analysis and writing – original draft. H.F.: project administration and writing – review and editing. J.Q.: funding acquisition, project administration, and writing – review and editing.

## Declaration of interests

The authors declare no competing interests.

## STAR★Methods

### Key resources table

**Table:** 

REAGENT or RESOURCE	SOURCE	IDENTIFIER
**Antibodies**		

NE	Abcam	Cat#ab254178; RRID: AB_3675863
LL-37	Abcam	Cat#ab69484; RRID: AB_2068681
MPO	ImmunoWay	Cat#YT5351; RRID: AB_3675865
cit-H3	Abcam	Cat#ab5103; RRID: AB_304752
GSDMD-N	ImmunoWay	Cat#YT 7991; RRID: AB_3663000
GSDMD	Servicebio	Cat#GB114198; RRID: AB_3675868

**Chemicals, peptides, and recombinant proteins**		

RNAlater	Thermo	Cat#AM7020
TRIzol	Thermo	Cat#15596026
Dextran T-500	Solarbio	Cat#D8270
Trypan Blue	Beyotime	Cat#ST2780
Wright's Stain	Beyotime	Cat#C0135
Giemsa Stain	Beyotime	Cat#C0133
RBC Lysis Buffer	Solarbio	Cat#R1010
PBS	CellMax	Cat#CBS101.05
Percoll	Sigma	Cat#P1644
RPMI1640 Medium	Thermo	Cat#11875093
DMEM	Thermo	Cat#C11995500BT
Fetal Bovine Serum	Biosharp	Cat#BIO-000007
PMA	Sigma	Cat#P8139
Chloroform	Macklin	Cat#10006818
Isopropyl Alcohol	Macklin	Cat#80109218
Anhydrous Ethanol	Macklin	Cat#E809056
Water Nuclease-Free	Servicebio	Cat#G4700
Goat Serum Blocking Solution	Solarbio	Cat#SL038
Xylene	Macklin	Cat#R017750
DAPI	Dawen	Cat#WBP823654
Antifade Mounting Medium	Servicebio	Cat#G1401-5ML
4% Paraformaldehyde	Beyotime	Cat#P0099
Neutral Mounting Medium	Solarbio	Cat#G8590
Citric Acid	Macklin	Cat#C805019
Hematoxylin Staining Solution	Beyotime	Cat#C0107
Eosin Staining Solution	Solarbio	Cat#G1100
Tris Buffered Saline with Tween	Solarbio	Cat#T1081

### Experimental model and study participant details

#### Human subjects

Samples from patients and healthy donors were taken following informed consent with ethical approval of the Clinical Research Ethics Committee of the First Affiliated Hospital, College of Medicine, Zhejiang University (Approval Number: 2022-IIT-554). We included a total of 11 patients with pyoderma gangrenosum (PG) and matched 11 healthy controls (HC) based on age and sex. The average age of the 11 patients was approximately 51 years, with 6 males and 5 females. Among the 11 patients, 2 had hematological malignancies, and 2 had inflammatory bowel disease. All patients received systemic glucocorticoid therapy, and biopsy and serum collection were performed before the initiation of glucocorticoid treatment. Additionally, serum collection was repeated in 4 patients after their conditions had improved.

#### Murine models

All mouse experiments were performed under protocols approved by the Tab of Animal Experimental Ethical Inspection of the First Affiliated Hospital, Zhejiang University School of Medicine (Approval Number: 2022–739). This experiment employed female C57BL/6J mice, aged between 8 and 12 weeks. Initially, a small electric grooming device was utilized to shave a region of 2 cm × 3 cm on the dorsum of the mice. Following this, an adequate amount of depilatory cream was evenly applied to induce depilation. The depilated area was then gently wiped with a saline-moistened gauze, revealing a smooth and hairless skin surface on the dorsum of the mice. After a resting period of 2 days, the mice, belonging to the PG group, were subjected to subsequent experiments. On the 0th, 1st, and 2nd days, 100 μL of serum (comprising 60% serum obtained from PG patients and 40% PBS) was subcutaneously injected into the same region of the mice's dorsal skin, resulting in noticeable wheal formation. The serum used to induce PG in mice was collected from PG patients before they received any treatment. Similarly, the mice in the HC group received subcutaneous injections of 100 μL of serum (comprising 60% serum obtained from HC subjects and 40% PBS) on the 0th, 1st, and 2nd days, which also led to the appearance of distinct wheals.

### Method details

#### Measurement of NETs

Serum NETs levels from PG and HC blood samples were detected by MPO-DNA ELISA kit (Hengyuan biochemical) according to the manufacturers’ instructions.

#### Immunofluorescence staining

Human and mice skin sections or isolated neutrophils were fixed with paraformaldehyde (4%). The samples were washed with TBST and serum blocked, and stained with primary antibodies overnight at 4°C. Next, the samples were incubated with followed by fluorochrome-conjugate secondary antibody and 4,6-diamidino-2-phenylindole staining. All images acquired were analyzed using Fiji by ImageJ.

### Quantification and statistical analysis

The analysis of data was conducted using SPSS statistical software and Prism software (GraphPad Software, Inc.). Categorical data was presented using frequencies and percentages, while comparisons between two groups were assessed using the chi-square test. For continuous data, the mean ± standard deviation or the median was used for representation. If the continuous data followed a normal distribution, the independent samples t-test was employed for comparing between two groups. However, if the data did not follow a normal distribution, the Mann-Whitney U test was used for the comparison. Data are represented as SEM and statistical significance as: ∗*p* < 0.05, ∗∗*p* < 0.01, and ∗∗∗*p* < 0.001, respectively.

## References

[1] Maverakis E., Marzano A.V., Le S.T., Callen J.P., Brüggen M.C., Guenova E., Dissemond J., Shinkai K., Langan S.M. (2020). Pyoderma gangrenosum. Nat. Rev. Dis. Primers.

[2] Brunsting L.A., Goeckerman W.H., O’Leary P.A. (1930). Pyoderma (echthyma) gangrenosum - clinical and experimental observations in five cases occurring in adults. Arch. Dermatol. Syphilol..

[3] Binus A.M., Qureshi A.A., Li V.W., Winterfield L.S. (2011). Pyoderma gangrenosum: a retrospective review of patient characteristics, comorbidities and therapy in 103 patients. Br. J. Dermatol..

[4] Romagnuolo M., Moltrasio C., Iannone C., Gattinara M., Cambiaghi S., Marzano A.V. (2023). Pyoderma gangrenosum following anti-TNF therapy in chronic recurrent multifocal osteomyelitis: drug reaction or cutaneous manifestation of the disease? A critical review on the topic with an emblematic case report. Front. Med..

[5] Nesterovitch A.B., Gyorfy Z., Hoffman M.D., Moore E.C., Elbuluk N., Tryniszewska B., Rauch T.A., Simon M., Kang S., Fisher G.J. (2011). Alteration in the gene encoding protein tyrosine phosphatase nonreceptor type 6 (PTPN6/SHP1) may contribute to neutrophilic dermatoses. Am. J. Pathol..

[6] Marzano A.V., Ceccherini I., Gattorno M., Fanoni D., Caroli F., Rusmini M., Grossi A., De Simone C., Borghi O.M., Meroni P.L. (2014). Association of pyoderma gangrenosum, acne, and suppurative hidradenitis (PASH) shares genetic and cytokine profiles with other autoinflammatory diseases. Medicine (Baltim.).

[7] Marzano A.V., Ishak R.S., Colombo A., Caroli F., Crosti C. (2012). Pyoderma gangrenosum, acne and suppurative hidradenitis syndrome following bowel bypass surgery. Dermatology.

[8] Marzano A.V., Damiani G., Ceccherini I., Berti E., Gattorno M., Cugno M. (2017). Autoinflammation in pyoderma gangrenosum and its syndromic form (pyoderma gangrenosum, acne and suppurative hidradenitis). Br. J. Dermatol..

[9] Braun-Falco M., Kovnerystyy O., Lohse P., Ruzicka T. (2012). Pyoderma gangrenosum, acne, and suppurative hidradenitis (PASH)--a new autoinflammatory syndrome distinct from PAPA syndrome. J. Am. Acad. Dermatol..

[10] Moura R.R., Brandão L., Moltrasio C., Agrelli A., Tricarico P.M., Maronese C.A., Crovella S., Marzano A.V. (2023). Different molecular pathways are disrupted in Pyoderma gangrenosum patients and are associated with the severity of the disease. Sci. Rep..

[11] Tallon B., Corkill M. (2006). Peculiarities of PAPA syndrome. Rheumatology.

[12] Alberts J.H., Sams H.H., Miller J.L., King L.E. (2002). Familial ulcerative pyoderma gangrenosum: a report of 2 kindred. Cutis.

[13] Khandpur S., Mehta S., Reddy B.S. (2001). Pyoderma gangrenosum in two siblings: a familial predisposition. Pediatr. Dermatol..

[14] al-Rimawi H.S., Abuekteish F.M., Daoud A.S., Oboosi M.M. (1996). Familial pyoderma gangrenosum presenting in infancy. Eur. J. Pediatr..

[15] Shands J.W., Flowers F.P., Hill H.M., Smith J.O. (1987). Pyoderma gangrenosum in a kindred. Precipitation by surgery or mild physical trauma. J. Am. Acad. Dermatol..

[16] Henry C.M., Sullivan G.P., Clancy D.M., Afonina I.S., Kulms D., Martin S.J. (2016). Neutrophil-Derived Proteases Escalate Inflammation through Activation of IL-36 Family Cytokines. Cell Rep..

[17] Takeuchi F., Sterilein R.D., Hall R.P. (2003). Increased E-selectin, IL-8 and IL-10 gene expression in human skin after minimal trauma. Exp. Dermatol..

[18] Maverakis E., van den Elzen P., Sercarz E.E. (2001). Self-reactive T cells and degeneracy of T cell recognition: evolving concepts-from sequence homology to shape mimicry and TCR flexibility. J. Autoimmun..

[19] Russell S.E., Horan R.M., Stefanska A.M., Carey A., Leon G., Aguilera M., Statovci D., Moran T., Fallon P.G., Shanahan F. (2016). IL-36alpha expression is elevated in ulcerative colitis and promotes colonic inflammation. Mucosal Immunol..

[20] Boutet M.A., Bart G., Penhoat M., Amiaud J., Brulin B., Charrier C., Morel F., Lecron J.C., Rolli-Derkinderen M., Bourreille A. (2016). Distinct expression of interleukin (IL)-36alpha, beta and gamma, their antagonist IL-36Ra and IL-38 in psoriasis, rheumatoid arthritis and Crohn's disease. Clin. Exp. Immunol..

[21] Hessam S., Sand M., Gambichler T., Skrygan M., Rüddel I., Bechara F.G. (2018). Interleukin-36 in hidradenitis suppurativa: evidence for a distinctive proinflammatory role and a key factor in the development of an inflammatory loop. Br. J. Dermatol..

[22] Smith E.J., Allantaz F., Bennett L., Zhang D., Gao X., Wood G., Kastner D.L., Punaro M., Aksentijevich I., Pascual V., Wise C.A. (2010). Clinical, Molecular, and Genetic Characteristics of PAPA Syndrome: A Review. Curr. Genomics.

[23] Weiss D.I., Ma F., Merleev A.A., Maverakis E., Gilliet M., Balin S.J., Bryson B.D., Ochoa M.T., Pellegrini M., Bloom B.R., Modlin R.L. (2019). IL-1beta Induces the Rapid Secretion of the Antimicrobial Protein IL-26 from Th17 Cells. J. Immunol..

[24] Marzano A.V., Borghi A., Meroni P.L., Cugno M. (2016). Pyoderma gangrenosum and its syndromic forms: evidence for a link with autoinflammation. Br. J. Dermatol..

[25] Senra L., Stalder R., Alvarez Martinez D., Chizzolini C., Boehncke W.H., Brembilla N.C. (2016). Keratinocyte-Derived IL-17E Contributes to Inflammation in Psoriasis. J. Invest. Dermatol..

[26] Senra L., Mylonas A., Kavanagh R.D., Fallon P.G., Conrad C., Borowczyk-Michalowska J., Wrobel L.J., Kaya G., Yawalkar N., Boehncke W.H., Brembilla N.C. (2019). IL-17E (IL-25) Enhances Innate Immune Responses during Skin Inflammation. J. Invest. Dermatol..

[27] Wang E.A., Steel A., Luxardi G., Mitra A., Patel F., Cheng M.Y., Wilken R., Kao J., de Ga K., Sultani H. (2017). Classic Ulcerative Pyoderma Gangrenosum Is a T Cell-Mediated Disease Targeting Follicular Adnexal Structures: A Hypothesis Based on Molecular and Clinicopathologic Studies. Front. Immunol..

[28] Antiga E., Maglie R., Volpi W., Bianchi B., Berti E., Marzano A.V., Caproni M. (2017). T helper type 1-related molecules as well as interleukin-15 are hyperexpressed in the skin lesions of patients with pyoderma gangrenosum. Clin. Exp. Immunol..

[29] Burn G.L., Foti A., Marsman G., Patel D.F., Zychlinsky A. (2021). The Neutrophil. Immunity.

[30] Lahoz-Beneytez J., Elemans M., Zhang Y., Ahmed R., Salam A., Block M., Niederalt C., Asquith B., Macallan D. (2016). Human neutrophil kinetics: modeling of stable isotope labeling data supports short blood neutrophil half-lives. Blood.

[31] Lawrence S.M., Corriden R., Nizet V. (2020). How Neutrophils Meet Their End. Trends Immunol..

[32] Mistry P., Carmona-Rivera C., Ombrello A.K., Hoffmann P., Seto N.L., Jones A., Stone D.L., Naz F., Carlucci P., Dell'Orso S. (2018). Dysregulated neutrophil responses and neutrophil extracellular trap formation and degradation in PAPA syndrome. Ann. Rheum. Dis..

[33] Kinoshita M., Ogawa Y., Hama N., Ujiie I., Hasegawa A., Nakajima S., Nomura T., Adachi J., Sato T., Koizumi S. (2021). Neutrophils initiate and exacerbate Stevens-Johnson syndrome and toxic epidermal necrolysis. Sci. Transl. Med..

[34] Li S., Ying S., Wang Y., Lv Y., Qiao J., Fang H. (2024). Neutrophil extracellular traps and neutrophilic dermatosis: an update review. Cell Death Discov..

[35] Pires R.H., Felix S.B., Delcea M. (2016). The architecture of neutrophil extracellular traps investigated by atomic force microscopy. Nanoscale.

[36] Petretto A., Bruschi M., Pratesi F., Croia C., Candiano G., Ghiggeri G., Migliorini P. (2019). Neutrophil extracellular traps (NET) induced by different stimuli: A comparative proteomic analysis. PLoS One.

[37] Silva C.M.S., Wanderley C.W.S., Veras F.P., Gonçalves A.V., Lima M.H.F., Toller-Kawahisa J.E., Gomes G.F., Nascimento D.C., Monteiro V.V.S., Paiva I.M. (2022). Gasdermin-D activation by SARS-CoV-2 triggers NET and mediate COVID-19 immunopathology. Crit. Care.

[38] Silva C.M.S., Wanderley C.W.S., Veras F.P., Sonego F., Nascimento D.C., Gonçalves A.V., Martins T.V., Cólon D.F., Borges V.F., Brauer V.S. (2021). Gasdermin D inhibition prevents multiple organ dysfunction during sepsis by blocking NET formation. Blood.

[39] Chen K.W., Monteleone M., Boucher D., Sollberger G., Ramnath D., Condon N.D., von Pein J.B., Broz P., Sweet M.J., Schroder K. (2018). Noncanonical inflammasome signaling elicits gasdermin D-dependent neutrophil extracellular traps. Sci. Immunol..

[40] Ortega-Loayza A.G., Friedman M.A., Reese A.M., Liu Y., Greiling T.M., Cassidy P.B., Marzano A.V., Gao L., Fei S.S., Rosenbaum J.T. (2022). Molecular and Cellular Characterization of Pyoderma Gangrenosum: Implications for the Use of Gene Expression. J. Invest. Dermatol..

[41] Croia C., Dini V., Loggini B., Manni E., Romanelli M., Migliorini P. (2021). Evaluation of neutrophil extracellular trap deregulated formation in pyoderma gangrenosum. Exp. Dermatol..

[42] Bonnekoh H., Scheffel J., Wu J., Hoffmann S., Maurer M., Krause K. (2019). Skin and Systemic Inflammation in Schnitzler's Syndrome Are Associated With Neutrophil Extracellular Trap Formation. Front. Immunol..

[43] Lukens J.R., Vogel P., Johnson G.R., Kelliher M.A., Iwakura Y., Lamkanfi M., Kanneganti T.D. (2013). RIP1-driven autoinflammation targets IL-1alpha independently of inflammasomes and RIP3. Nature.

[44] Croker B.A., Lawson B.R., Rutschmann S., Berger M., Eidenschenk C., Blasius A.L., Moresco E.M.Y., Sovath S., Cengia L., Shultz L.D. (2008). Inflammation and autoimmunity caused by a SHP1 mutation depend on IL-1, MyD88, and a microbial trigger. Proc. Natl. Acad. Sci. USA.

[45] Mazgaeen L., Yorek M., Saini S., Vogel P., Meyerholz D.K., Kanneganti T.D., Gurung P. (2023). CD47 halts Ptpn6-deficient neutrophils from provoking lethal inflammation. Sci. Adv..

[46] Demkow U. (2023). Molecular Mechanisms of Neutrophil Extracellular Trap (NETs) Degradation. Int. J. Mol. Sci..

[47] Tang S., Yang C., Li S., Ding Y., Zhu D., Ying S., Sun C., Shi Y., Qiao J., Fang H. (2022). Genetic and pharmacological targeting of GSDMD ameliorates systemic inflammation in macrophage activation syndrome. J. Autoimmun..

