# Supplementary material for: Gasdermin D-dependent neutrophil extracellular traps exacerbate cytokine storm contributing to pyoderma gangrenosum pathogenesis
Source: iScience. 2025 Jan 30;28(3):111925. doi: 10.1016/j.isci.2025.111925 (PMC11872606; doi:10.1016/j.isci.2025.111925)
Supplement: Document S1. Figures S1–S3 [file mmc1.pdf]

iScience, Volume *28*

Supplemental information

Gasdermin D-dependent neutrophil extracellular traps exacerbate cytokine storm contributing to pyoderma gangrenosum pathogenesis

Sheng Li, Shuni Ying, Hong Fang, and Jianjun Qiao

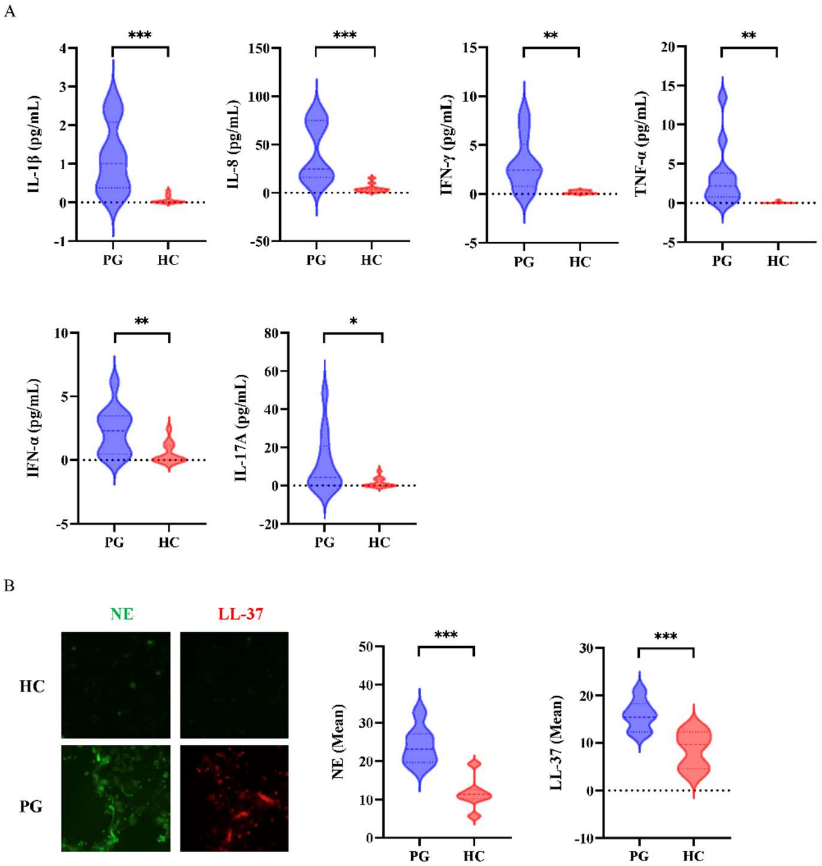

Fig. S1. Cytokine expression levels in Serum and NET formation in neutrophils of PG patients.

(A) The expression levels of IL-1β, IL-8, IL-17A, IL-6, IL-10, TNF-α, IFN-γ, and IFN-α in the serum of PG patients. (B) The expression levels of NETs in neutrophils extracted from the peripheral blood of PG patients. IL: interleukin; PG: pyoderma gangrenosum; TNF: tumor necrosis factor; IFN: interferon. *$p < 0.05$, **$p < 0.01$, and ***$p < 0.001$, respectively.

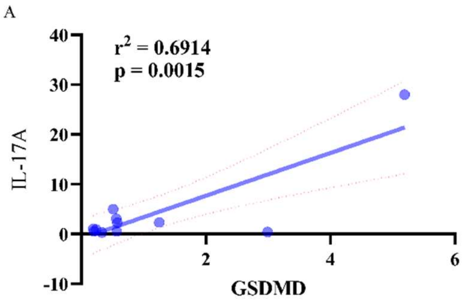

Fig. S2. Correlation between GSDMD expression in serum and IL-17A expression in

PG patients.

(A) The correlation of the expression level of GSDMD in the serum of PG patients and the expression level of IL-17A. IL: interleukin; PG: pyoderma gangrenosum; GSDMD: gasdermin D.

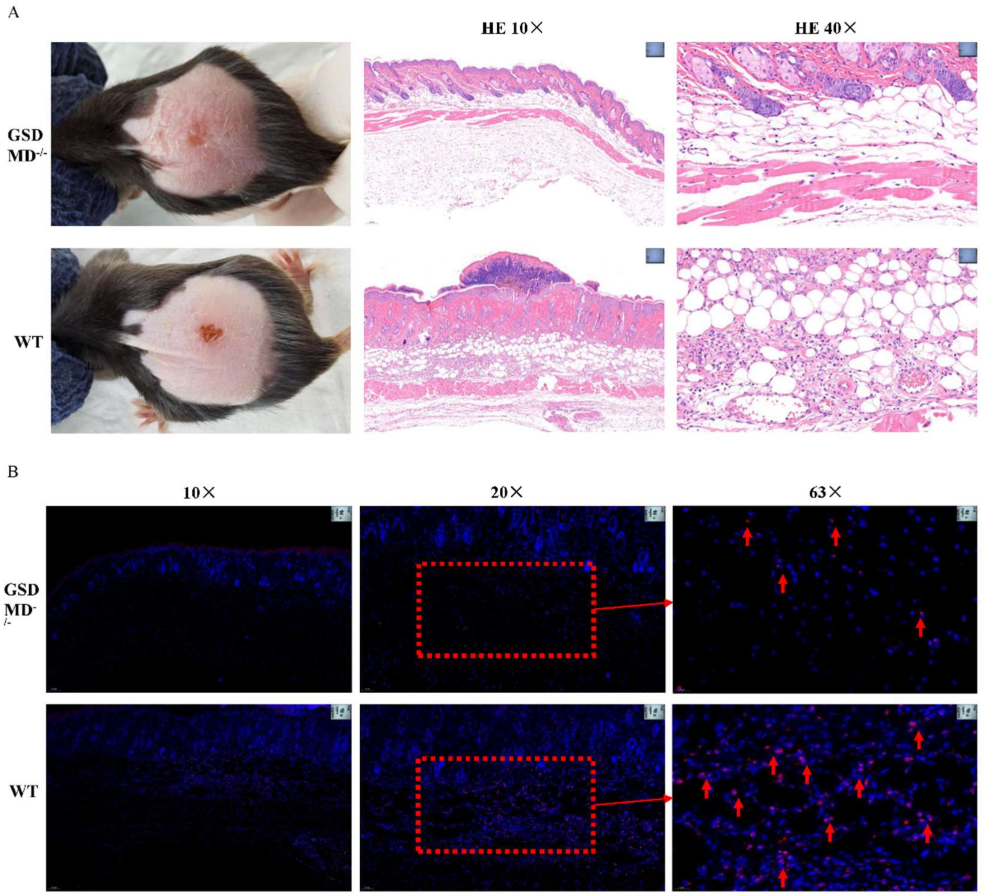

Fig. S3. Histopathological features and MPO expression in skin lesions of *GSDMD*$^{-/-}$ mice compared to WT mice.

(A) The histopathological features of the skin lesions in the *GSDMD*$^{-/-}$ group mice compared to WT mice group mice. (B) The expression level of MPO of the skin lesions in the *GSDMD*$^{-/-}$ group mice compared to WT mice group mice. WT: wild type; GSDMD: gasdermin D; MPO: myeloperoxidase.
